# Early Evolution of Transcription Systems and Divergence of Archaea and Bacteria

**DOI:** 10.3389/fmolb.2021.651134

**Published:** 2021-05-05

**Authors:** Lei Lei, Zachary F. Burton

**Affiliations:** ^1^Department of Biology, University of New England, Biddeford, ME, United States; ^2^Department of Biochemistry and Molecular Biology, Michigan State University, E. Lansing, MI, United States

**Keywords:** archaea, bacteria, double-Ψ-β-barrel, general transcription factor evolution, promoter evolution, transcription, transcription factor B, sigma factor

## Abstract

DNA template-dependent multi-subunit RNA polymerases (RNAPs) found in all three domains of life and some viruses are of the two-double-Ψ-β-barrel (DPBB) type. The 2-DPBB protein format is also found in some RNA template-dependent RNAPs and a major replicative DNA template-dependent DNA polymerase (DNAP) from Archaea (PolD). The 2−DPBB family of RNAPs and DNAPs probably evolved prior to the last universal common cellular ancestor (LUCA). Archaeal Transcription Factor B (TFB) and bacterial σ factors include homologous strings of helix-turn-helix units. The consequences of TFB-σ homology are discussed in terms of the evolution of archaeal and bacterial core promoters. Domain-specific DPBB loop inserts functionally connect general transcription factors to the RNAP active site. Archaea appear to be more similar to LUCA than Bacteria. Evolution of bacterial σ factors from TFB appears to have driven divergence of Bacteria from Archaea, splitting the prokaryotic domains.

## Introduction

The purpose of this review is to provide a conceptual overview of transcription systems in the early phase of their evolution, in order to explain how RNA polymerases (RNAPs), general transcription factors and promoters may have evolved. The review also touches on the divergence of Archaea and Bacteria that appears to have partly been driven by the divergence of transcription systems. The proper way to view structures is using molecular graphics such as UCSF ChimeraX (Goddard et al., 2018; Pettersen et al., 2021). Viewing structures in 2−dimensions is challenging to the human eyes and mind. We recommend downloading ChimeraX, running tutorials and using it to follow along with this manuscript. For instance, some figures in this paper are difficult to fully appreciate without a more 3-dimensional representation.

Our opinion is that analyzing the structure-function-dynamics of any protein requires a combination of approaches: i.e., (1) structure analysis; (2) evolution; (3) functional studies; and (4) dynamics. To appreciate structural analysis and dynamics, Cryo-electron microscopy becomes an ever more powerful tool. Cryo-EM provides ensembles of structures often indicating a dynamic progression through a reaction mechanism. Evolutionary studies have the potential to dissect a protein into its component parts to better appreciate how the protein came to have its eventual form and function. In some cases, structural studies have not been combined fully with evolutionary studies, and the historic naming of protein domains can be confusing. Also, very large structures are difficult to analyze unless they can be broken into component parts. We see two potential problems. Without an evolutionary view, structures may be difficult to understand and analyze. Also, the evolution literature can be complex and challenging to read unless one is reasonably expert or determined. In this paper, we attempt to apply a combination of structural and evolutionary principles to the analysis and description of multi-subunit RNAPs, general transcription factors and promoters.

## Evolution of 2-DPBB RNAPs and DNAPs

### 2-Double-Ψ-β-Barrel Type RNAPs

**FIGURE 1:**
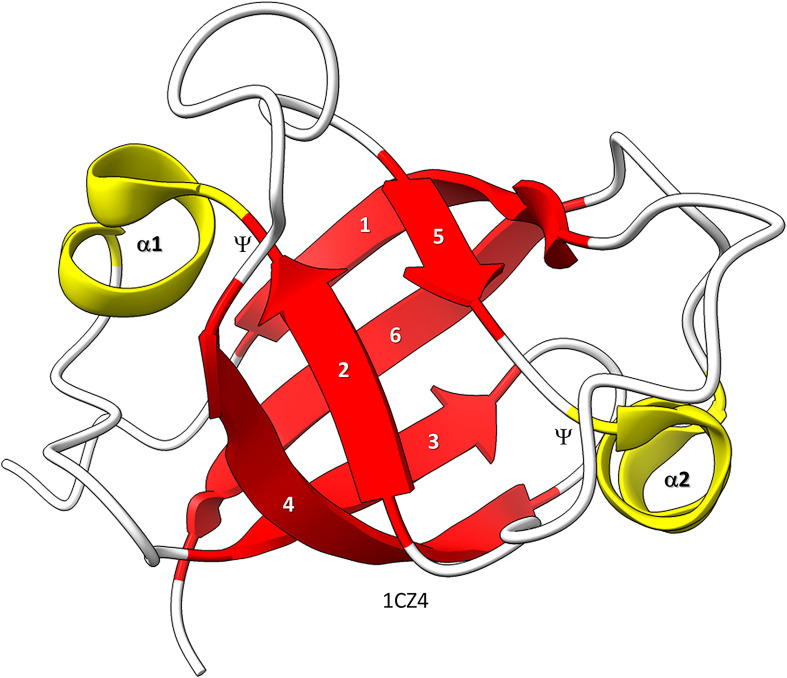
Bacterial VAT (VCP−like ATPase) includes a simple DPBB. ChimeraX was used for molecular graphics (Goddard et al., 2018; Pettersen et al., 2021). The structure is PDB 1CZ4 (Coles et al., 1999). β−sheets are red; α−helices are yellow. Ψ indicates the Ψ pattern of crossing peptide chains.

**FIGURE 2:**
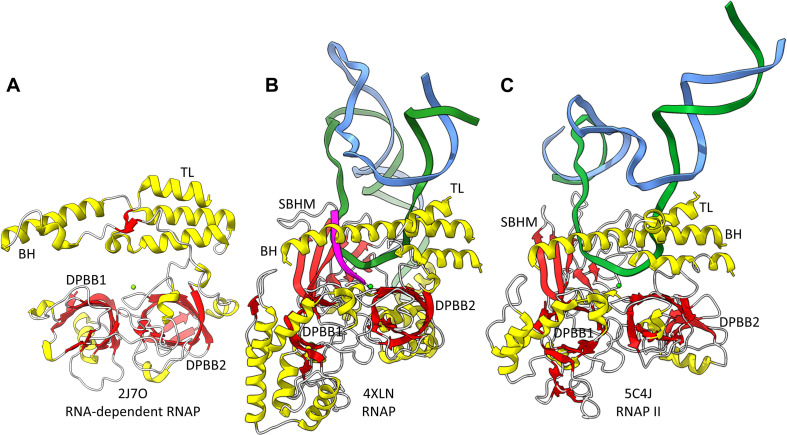
The catalytic core of 2-DPBB type RNAPs. **(A)** A RNA template-dependent RNAP from *Neurospora crassa* (PDB 2J7O) (Salgado et al., 2006). **(B)** A bacterial multi-subunit RNAP (PDB 4XLN) (Bae et al., 2015). **(C)** A human multi-subunit RNAP (PDB 5C4J) (Barnes et al., 2015). α-helices are yellow; β-sheets are red; Mg is green; RNA is magenta; template DNA is green; non-template DNA is blue. BH indicates the bridge helix. TL indicates the trigger loop. The active site is identified by the Mg (Mg1) and the 3′-end of the RNA **(B,C)**.

Near the dawn of evolution of life on Earth, RNAPs of the 2-DPBB type evolved (Iyer et al., 2003; Lane and Darst, 2010a,b; Werner and Grohmann, 2011; Iyer and Aravind, 2012; Fouqueau et al., 2017; Sauguet, 2019; Madru et al., 2020; Zatopek et al., 2020). These enzymes are found in all domains of life and some viruses. 2-DPBB RNAPs can be either RNA template-dependent or DNA template-dependent, indicating that this important class of enzyme may have arisen in an RNA world before DNA genomes became prominent. The DPBB is a particular fold of cradle-loop barrel (Figure 1; Coles et al., 2005, 2006; Alva et al., 2008). The crossing chains make a Ψ pattern, hence the barrel name. 2−DPBB type RNAPs have 2-DPBBs at their active sites (Figure 2). Loops from the barrels hold the two Mg^2+^ that retain the phosphates of the NTP substrate and activate the RNA 3′-O to catalyze NMP addition. In addition to the 2-DPBBs, both RNA and DNA template-dependent RNAPs have a bridge helix and trigger loop, indicating that these elements are ancient (Salgado et al., 2006; Iyer and Aravind, 2012; Qian et al., 2016). In DNA template-dependent RNAPs, the β-subunit DPBB1 has a sandwich-barrel hybrid motif (SBHM) inserted into one of the barrel loops (Lane and Darst, 2010a,b; Iyer and Aravind, 2012; Fouqueau et al., 2017). The SBHM loop extension forms the historically-named “flap” or “wall” motif in multi-subunit RNAPs.

Barrels are frequent motifs in ancient evolution. In earliest evolution, barrels were selected to form compact, structured units with reasonable solubility and structural closure (Burton et al., 2016). For instance, 8−β-sheet barrels [(β−α)_8_; i.e., TIM barrels (TIM for triose phosphate isomerase)] are found in most glycolytic enzymes. Rossmann folds appear to be sheets that are rearranged from (β − α)_8_ barrels. Most of the citric acid cycle is made up of Rossmann fold proteins. So, much of core metabolism was generated from barrels and, also, from refolded barrels rendered into more linear sheets. Cradle-loop barrels are a similar ancient evolution story (Alva et al., 2008). If early evolution was partly a race to form stable and soluble scaffolds, formation of barrels helped to build these and, among other possible advantages, helped to avoid generation of β−sheet amyloids and liquid-liquid phase separated compartments that resisted ordered protein folding. Clearly, barrels were a successful evolutionary innovation that, once formed, persisted throughout evolution. From this point of view, an important evolutionary event can be viewed as the race to form stable and soluble protein structures with a degree of structural closure. Barrels were typically formed in evolution by repeated motif duplications, so barrels often won races to higher order structure, solubility and closure. After generation of barrels, primitive catalytic sites could be modified to generate many new, more efficient and more specific enzyme functions. So, for instance, in metabolism, an enzyme with broad specificity built around an 8−β-sheet barrel was duplicated genetically many times and then refined, generating specialist enzymes that formed a more sophisticated and integrated pathway (i.e., glycolysis).

Similarly, the DPBB evolved by duplication of a β − β − α − β unit followed by refolding into a barrel (Alva et al., 2008; Burton et al., 2016). In Figure 1, a β − β − α − β − − β − β − α − β DPBB enzyme domain is shown in which the basic DPBB form is preserved without much modification (Coles et al., 1999). The β−sheets are numbered 1−6, so that the chain can be traced. The α−helices are numbered 1 and 2. The Ψ patterns of the crossing chains are indicated. The ability to identify a DPBB helps with understanding the 2−DPBB enzyme patterns when analyzing more complex structures. Because of modifications of the pattern during evolution or disorder in structures, DPBBs can be a challenge to identify and, in a complex structure, can be potentially difficult to locate.

**FIGURE 3:**
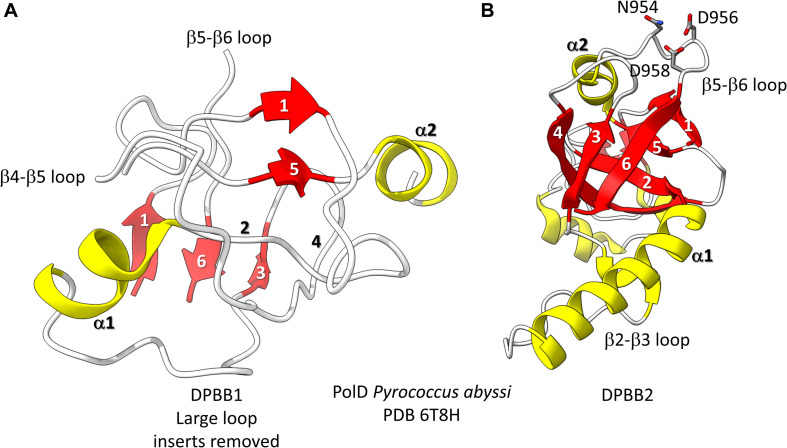
The two DPBBs of a DNA template-dependent DNAP (archaeal PolD) (PDB 6T8H) (Madru et al., 2020). Colors are as in Figures 1, 2. **(A)** DPBB1 is somewhat disordered in the structure, so not all β-sheets were scored as such by ChimeraX. In **(B)** DPBB2, N954, D956 and D958 may hold the active site Mg (missing in the structure) (Zatopek et al., 2020).

2−DPBB type enzymes include RNA template−dependent RNAPs (found in some Eukaryotes), multi−subunit RNAPs (found in all domains and some viruses) and DNA template−dependent DNAPs (PolD in most Archaea) (Figures 2, 3; Iyer et al., 2003; Lane and Darst, 2010a,b; Werner and Grohmann, 2011; Iyer and Aravind, 2012; Fouqueau et al., 2017; Koonin et al., 2020). In 2−DPBB type enzymes, the basic β − β − α − β − − β − β − α − β form can be modified by insertions into barrel loops. In RNA template-dependent 2−DPBB RNAPs, neither DPBB1 (corresponding to the β−subunit DPBB1 in 2−DPBB bacterial RNAPs) nor DPBB2 (corresponding to the β′-subunit DPBB2 in 2−DPBB bacterial RNAPs) includes very large inserts or modifications in the basic DPBB pattern (Salgado et al., 2006; Iyer and Aravind, 2012; Qian et al., 2016).

In DNA template-dependent 2−DPBB type RNAPs, by contrast, there are large identifying inserts (Iyer and Aravind, 2012). Significantly, the β−subunit (referring to bacterial RNAPs) DPBB1, includes a sandwich-barrel hybrid motif (SBHM) inserted between β2 and β3 after α1. The SBHM can be recognized because it includes long β−sheets. The SBHM forms the “flap” or “wall” domain of the RNAP that contacts σ (Bacteria) and TFB (Archaea) general transcription factors. The SBHM also contacts the general elongation factors NusG (Bacteria) and Spt5/Spt4 (Archaea). Because the SBHM is missing in RNA template−dependent RNAPs of the 2−DPBB type, the SBHM is considered to be a feature for the transcription of DNA templates (Iyer and Aravind, 2012). Because the SBHM interacts with initiation factors, the SBHM is considered to be evolved to facilitate initiation from DNA templates. A large mostly α−helical insert is found between DPBB1 β5 and β6, after α2. This insert is only partially homologous in archaeal and bacterial RNAPs and appears to make domain-specific contacts to RNAP rather than contacts to transcription factors. In some structures, DPBB1 is somewhat disordered in 2−DPBB DNA template-dependent RNAPs, making some of the β−sheets difficult to discern. The β′-subunit DPBB2 (referring to bacterial RNAPs) has a largely α−helical insert between β2 and β3 (distinct from the SBHM that includes long β−sheets). In Archaea, the insert between DPBB2 β2 and β3 is referred to as a RAGNYA domain that includes β−sheets and α−helices (Balaji and Aravind, 2007; Iyer and Aravind, 2012). The archaeal and bacterial DPBB2 β2−β3 inserts are very different in sequence and make domain-specific contacts to TFB and σ for initiation.

Found in many Archaea, PolD are DNA template-dependent DNAPs of the 2−DPBB form engaged in genomic replication (Raia et al., 2019; Sauguet, 2019; Koonin et al., 2020; Madru et al., 2020). In these enzymes DPBB1 includes two large inserts, one between β4 and β5 and one between β5 and β6. In available structures, PolD DPBB1 appears to be somewhat disordered, similarly to DPBB1 (β−subunit of bacterial RNAPs) in some structures of DNA template-dependent RNAPs. The significance of this possible similarity in some structures is not known to us. One idea is that DPBB1 is somewhat more dynamic because it accommodates to the presence and absence of substrate to a larger extent than DPBB2, which holds active site Mg1 more tightly than DPBB1 holds Mg2. We would be interested to know whether dNTP binding tightens the PolD DPBB1 and whether similar changes might occur in multi-subunit RNAPs with NTP binding. In PolD, DPBB2 includes an insert between β1 and β2. The inserts in the DNA template-dependent DNAPs (PolD) discriminate PolD enzymes from multi-subunit RNAPs and RNA template-dependent RNAPs and indicate how these more complex enzymes diverged from RNA template-dependent RNAPs of the 2-DPBB form (Koonin et al., 2020).

The story of evolution of these ancient 2-DPBB-type enzymes cannot now be told with certainty, but we construct a possible narrative. We posit that RNA template-dependent RNAPs may have evolved in an RNA-dominated world prior to LUCA (Iyer and Aravind, 2012; Koonin et al., 2020). These enzymes include no large inserts in their DPBBs, indicating that RNA template-dependent RNAPs probably comprise the most ancient 2-DPBB enzyme form. DNA template-dependent RNAPs (multi-subunit RNAPs) and DNAPs (PolD) appear to have radiated mostly independently from the primitive form, although, multi-subunit RNAPs and PolD may share one or two Zn motifs that are missing from 2-DPBB RNA template-dependent RNAPs (see below). Multi-subunit RNAPs and Pol D, however, have distinct DPBB loop inserts. To our knowledge, comparative sequence analyses of these enzymes provides limited insight into details of their divergence, because sequences among enzyme classes are only weakly conserved (Sauguet, 2019; Madru et al., 2020; Zatopek et al., 2020). Because PolD is ancient, this 2-DPBB type enzyme may be the initial evolved DNA template-dependent DNAP for genomic replication (i.e., at LUCA), and other DNAPs, i.e., PolA, PolB and PolC, may have evolved later (Koonin et al., 2020).

RNA template-dependent RNAPs and multi-subunit RNAPs have a recognizable bridge helix and trigger loop (Figure 2), and these features are altered and rearranged in DNA template-dependent DNAPs (PolD) of the 2-DPBB type (see below) (Madru et al., 2020). It appears that 2-DPBB multi-subunit RNAPs from Archaea and Eukaryotes and PolD from Archaea may share a Zn-finger motif that is missing from RNA template-dependent RNAPs and bacterial multi-subunit RNAPs. We posit that Archaea are older than Bacteria and closer to LUCA (Battistuzzi et al., 2004; Lei and Burton, 2020; Long et al., 2020), but also see Forterre (2015), Da Cunha et al. (2017, 2018), Castelle and Banfield (2018), Eme et al. (2018). We, therefore, posit that this Zn-finger was lost in bacterial multi-subunit RNAPs, which appear to be a simplified form compared to archaeal multi-subunit RNAPs. We posit that bacterial RNAPs were driven to diverge from archaeal RNAPs primarily because bacterial RNAPs co-evolved with bacterial σ factors.

### RNAP Catalytic Subunits (A Guided Tour)

Our view is that Archaea are older than Bacteria, and, therefore, Archaea are closer to LUCA (Battistuzzi et al., 2004; Marin et al., 2017; Lei and Burton, 2020; Long et al., 2020). For other views, see Forterre (2015), Da Cunha et al. (2017, 2018), Castelle and Banfield (2018), Eme et al. (2018). Because of horizontal gene transfer, some phylogenetic analyses may be misleading in determining the deep branching of prokaryotic domains. We believe Bacteria were derived from Archaea. Our opinions are based on ancient evolution studies of transcription systems, tRNA, aminoacyl-tRNA synthetases, ribosomes and the genetic code. In every comparison we have made, Archaea appear to be the more ancient lineage, and Bacteria appear to be more innovated and more derived evolutionarily from root sequences. Therefore, to describe the multi-subunit RNAP catalytic subunits, we use an archaeal RNAP as the example. The RNAP we selected is from *Saccharolobus shibatae* (PDB 2WB1) (Korkhin et al., 2009). The catalytic subunits include 2WB1_A and 2WB1_C (_A and _C indicates the chain designation), which correspond to the β′ subunit of bacterial RNAP, a subunit that is split in some Archaea. 2WB1_B corresponds to the β subunit of bacterial RNAPs. We compare similar motifs in DNAP PolD to emphasize early evolution of RNAPs.

**FIGURE 4:**
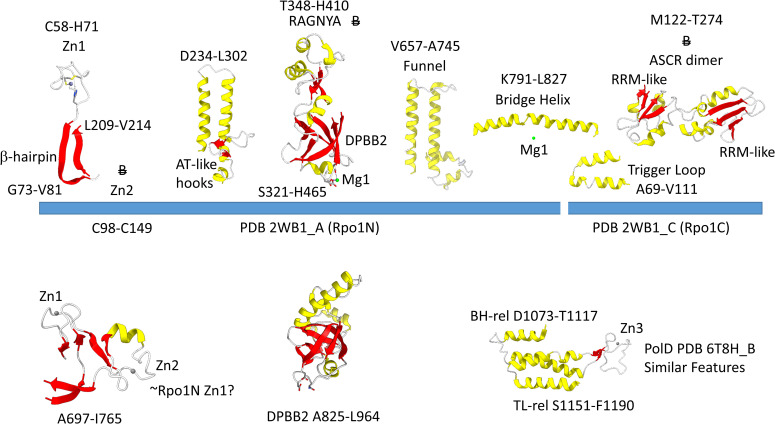
Some recognizable motifs that characterize the RpoA′ and RpoA″ subunits of archaeal RNAP, corresponding to the β′ subunit of bacterial RNAP (Korkhin et al., 2009). “B” with a double strike through indicates a motif in archaeal RNAP that is not identified in bacterial RNAP. Similar motifs in DNAP PolD are shown below the blue bar.

Figure 4 shows the Rpo1N (2WB1_A; A′) and Rpo1C (2WB1_C; A″) chains. We describe some recognizable protein motifs, reading from the N-terminus of the 2WB1_A chain through the 2WB1_C chain. Zn1 is very close to the 2WB1_A N-terminus. Evolutionarily-related motifs in PolD are indicated below the blue bar. Zn1 in 2WB1_A may correspond to archaeal DNAP PolD Zn2, based on its position in the structure and its distance from a Zn motif in chain 2WB1_B (Madru et al., 2020). The N-terminal β-sheet of the β-hairpin is next, followed by 2WB1_A Zn2, which is missing in bacterial RNAP. Next is the C-terminal β-hairpin. From D234 to L302 is a helix-loop-helix motif that connects the AT-like hooks (Iyer and Aravind, 2012). The AT-like hook loop contacts single-stranded DNA in the RNAP open complex and elongation complex. Next is the DPBB2 barrel. Between DPBB2 β2 and β3 after α1 is the RAGNYA insert. In Bacteria, a DPBB2 β2-β3 insert after α1 shows no detectable homology and is primarily α-helical (see below). DPBB2 holds Mg1 within the loop between DPBB2 β5 and β6 (NADFDGD). The “funnel” is located in the primary sequence between the DPBB2 and bridge helix. In the open transcription complex or elongation complex, the DNA template bends by about 90° and DNA strands separate over the bridge helix. DNA PolD has a similar DPBB2 and, also, modified structures that are probably genetically related to the bridge helix and trigger loop, although these features in PolD appear to be rearranged and repurposed (see below).

The *Saccharolobus shibatae* RNAP is separated into two genes relative to the bacterial RNAP β′ subunit, and the subunit separation is between the bridge helix and the trigger loop. The trigger loop is near the archaeal Rpo1C subunit (2WB1_C) N-terminus. The RNAP trigger loop appears to correlate with the PolD “clamp” structure (PDB 6T8H_B; S1151-F1190) (Madru et al., 2020). Near the C-terminus of archaeal RNAP Rpo1C, the ASCR dimer is located, with two RRM-like features (RRM for RNA-recognition motif) (Iyer and Aravind, 2012). The ASCR dimer motif is missing in bacterial RNAP and may have been lost by deletion.

**FIGURE 5:**
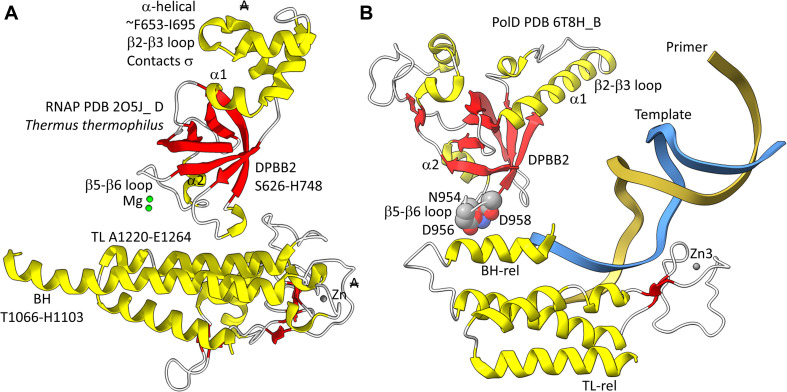
Similarities between the DPBB2, bridge helix and trigger loop of bacterial RNAP and related motifs in DNAP PolD. **(A)** Bacterial RNAP features. **(B)** Related PolD features. The similarly placed Zn motifs are not thought to be homologous. “A” with a double strike through indicates that a feature of bacterial RNAP is not present in archaeal RNAP.

**FIGURE 6:**
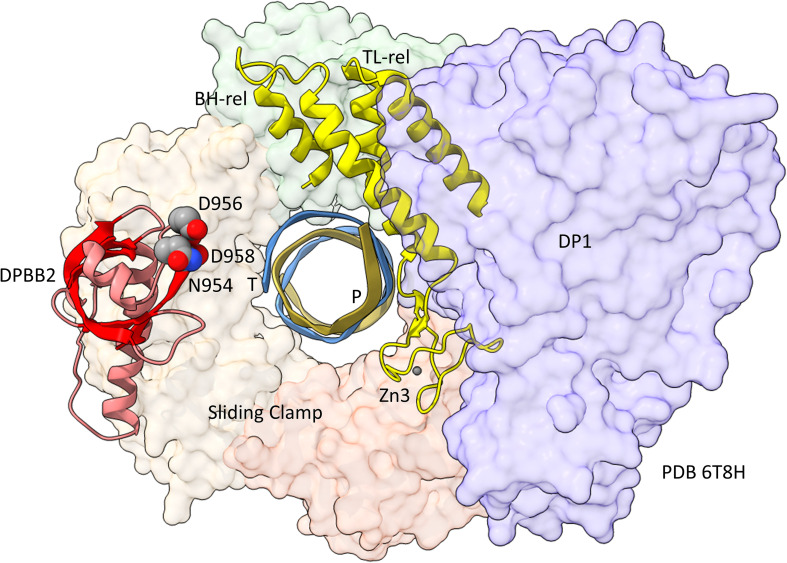
Repurposing of the bridge helix-related (BH-rel) and trigger loop-related (TL-rel) motifs in PolD. The DPBB2 (light red with red β-sheets) and BH-rel, Zn3 and TL-rel region (yellow) is shown for the DP2 2-DPBB subunit. (T) template DNA (blue); (P) primer DNA (gold). The sliding clamp trimer is shown (green, beige and orange). The DP1 subunit is blue. Active site residues that hold Mg1 are indicated in space-filling representation.

In Figure 5, a comparison is shown of bacterial RNAP DPBB2, the bridge helix and the trigger loop (Figure 5A) and related features in DNAP PolD (Figure 5B). In Figure 5A, an α-helical domain separates DPBB2 β2 and β3. The α-helical loop insert corresponds to and may have replaced the RAGNYA region in archaeal RNAP (Figure 4). The bacterial RNAP β′ subunit includes a Zn motif separating the bridge helix and the trigger loop that is missing in Archaea (Figure 5A). PolD also has a Zn motif (Zn3) separating its bridge helix-related and trigger loop-related features, although we do not think these Zn motifs in bacterial RNAP and PolD are related by homology. Rather these Zn motifs may be the result of convergent evolution. In bacterial RNAP, the trigger loop is closer to the active site than the bridge helix and closes over the NTP substrate to expel water from the active site and tighten the substrate for addition to the RNA chain (Vassylyev et al., 2007b). In the image in Figure 5A, the trigger loop is in the closed and catalytic conformation. In PolD, the trigger loop-related feature is further from the active site than the bridge helix-related feature. In PolD, the bundle of C-terminal α-helices (bridge helix-related and trigger loop-related features) bind DNA and, also, the proofreading PolD subunit (DP1; the 2-DPBBs are part of the DP2 subunit) (Figure 6). The DP1 subunit includes an exonuclease domain. Loops from the bridge helix-related and trigger loop-related PolD features also contact the sliding clamp that maintains PolD processivity (Madru et al., 2020). It appears, therefore, that, although bridge helix- and trigger loop-related features in PolD and RNAPs may be related by evolution, they fulfill different roles.

**FIGURE 7:**
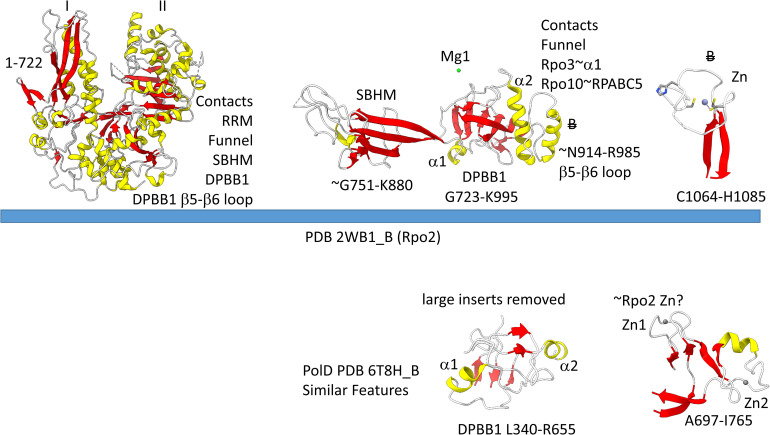
Some recognizable motifs in the Rpo2 subunit of archaeal RNAP (corresponding to the β subunit of bacterial RNAP) (Korkhin et al., 2009). Colors and abbreviations are as in Figure 4. Related motifs in DNAP PolD are indicated beneath the blue bar. “B” with a double strike through indicates a feature in Archaea that is missing or very different in Bacteria.

The archaeal RNAP Rpo2 subunit corresponds to the β-subunit in bacterial RNAP. Features of the Rpo2 RNAP subunit (PDB 2WB1_B; B) are shown in Figure 7. There is a 2-lobed N-terminal domain extending from position 1–722. The DPBB1 extends from G723 to K995. There are two notable inserts in DPBB1 loops. Between β2 and β3, just after α1, a SBHM is inserted (Iyer and Aravind, 2012). The SBHM is characterized by long β-sheets. In archaeal RNAP, the SBHM is referred to as the “wall” domain, which interacts with the general transcription factor TFB. In bacterial RNAP, the SBHM has been referred to as the “flap” domain, which interacts with the bacterial σ factor. Between β5 and β6, just after α2, an α-helical segment is inserted (∼N914-R985). At the C-terminus of the Rpo2 chain, a Zn finger is located in archaeal RNAPs but missing in bacterial RNAPs. Although the sequences are different, this Zn finger may correspond to Zn1 in archaeal DNAP PolD (Madru et al., 2020). As in PolD, the Rpo2 Zn finger and the Rpo1N Zn1 are close in space in archaeal RNAP, similar to PolD Zn1 and Zn2.

The description of the catalytic subunits of multi-subunit RNAPs here is incomplete. The intention is to provide some visible and conceptual guide posts for researchers as they begin to probe and familiarize themselves with RNAP structures. Also, we emphasize features that appear most important for interactions between general transcription factors and the RNAP catalytic center (see below). A more detailed description of RNAP evolution and domains is provided by Iyer and Aravind (2012). Reviews of the subunit structures of multi-subunit RNAPs are also published elsewhere (Jun et al., 2011; Osman and Cramer, 2020).

### 2-Mg Mechanism of Transcription by Multi-Subunit RNAPs

**FIGURE 8:**
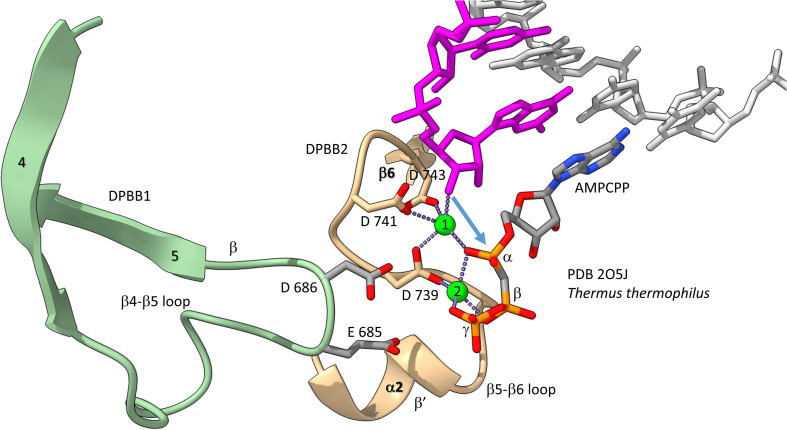
The two Mg mechanism for transcription by RNAP. The structure (PDB 205J) is from *Thermus thermophilus* (Vassylyev et al., 2007b). Mg1 and Mg2 (green spheres) are labeled. The RNA chain is magenta. The β′ subunit is beige. The β subunit is lime. Some active site residues are labeled. AMPCPP (a non-hydrolyzable substrate) is in the substrate site.

We have described the basic catalytic core of multi-subunit RNAPs: 2-DPBBs, a bridge helix and a trigger loop (Figures 2B,C). These enzymes utilize a 2-Mg mechanism for transcription (Figure 8; Vassylyev et al., 2007b). The 2-Mg (Mg1 and Mg2) are held by acidic groups (E and D) on loops of the 2-DPBBs. DPBB1 includes 685-ED-686 (*Thermus thermophilus* RNAP numbering) located on the DPBB1 loop between β4 and β5. D686 appears to interact with Mg2 during phosphodiester bond formation. Mg2 is loosely held in the RNAP structure. DPBB2 includes the highly conserved sequence 737-NADFDGD-743 within the loop between β5 and β6. D739, D741 and D743 strongly hold Mg1. It is thought that Mg1 remains bound to RNAP, but Mg2 may exchange with each NTP addition. Mg2 normally enters the RNAP bound to the NTP as NTP-Mg. The NADFDGD motif in multi-subunit RNAPs corresponds to 954-NCDGDED-961 in archaeal *Pyrococcus abyssi* DNAP PolD (Madru et al., 2020), although, in PolD, the active site Mg1 is held by N954, D956 and D958, so the Mg1-contacting residues are slightly shifted in PolD (Zatopek et al., 2020). In *Neurospora crassa* RNA template-dependent RNAP, Mg1 is held by 1005-GGDYDGD-1011 (Salgado et al., 2006; Qian et al., 2016). Acidic groups retaining Mg1 at the active enzyme site are highly conserved in 2-DPBB type enzymes, although PolD has slightly shifted the set of interacting residues. In the simplest cradle loop barrel enzymes, similar acidic groups can be identified in the same DPBB location (just before β3 and β6), indicating that the initial evolution of DPBBs may have been to chelate Mg (Coles et al., 1999).

Figure 8 shows the 2-Mg mechanism for RNA polymerization. The 3′-O of the RNA chain attacks the α-phosphate of the incoming NTP substrate to add a single NMP unit to the chain and to release pyrophosphate (Vassylyev et al., 2007a,b). Mg1 is held tightly by D739, D741 and D743 within the NADFDGD loop between β5 and β6 of the DPBB2 (β′subunit). Mg2 enters with the NTP substrate and probably interacts with D686 of the DPBB1 (β subunit). Mg2 probably leaves with pyrophosphate.

## Evolution of Archaeal and Bacterial GTFs

**FIGURE 9:**
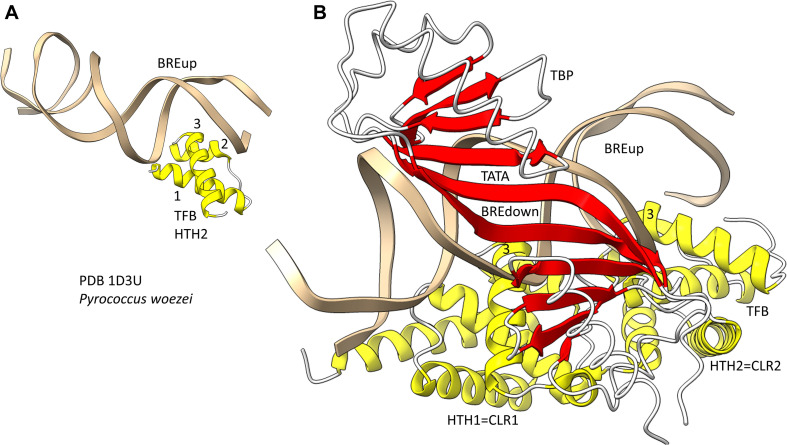
The promoter-TBP-TFB complex in Archaea. **(A)** A detail of the image in panel **(B)**, showing that TFB HTH units are typical and make typical contacts to the major groove of DNA. **(B)** The promoter-TBP-TFB complex. HTH1 and HTH2 are the last 3 helices of 5-helix cyclin-like repeats (CLR1 and CLR2).

Because we posit that Archaea are older than Bacteria, we first consider general transcription factors (GTFs) in Archaea (Jun et al., 2011; Blombach et al., 2015). To recognize a core promoter, Archaea utilize TBP (TATA-box binding protein), TFB (transcription factor B) and TFE (transcription factor E). It appears that Bacteria evolved σ factors from TFB and lost TBP and TFE in evolution. Figure 9 shows a promoter-TBP-TFB complex from Archaea (Littlefield et al., 1999). Figure 9A is a detail of the image in Figure 9B to indicate the helix-turn-helix (HTH) motif of the most C-terminal HTH domain. TBP contacts the 8-nt TATA-box. TBP includes a C-terminal repeat sequence that forms a pseudo-dimer of β-sheet folds to align with pseudo-dimeric DNA. TBP occupies the minor groove of the DNA. TFB includes two cyclin-like repeats (CLR) formed as 5-α-helix bundles that bind DNA upstream and downstream of TATA (Lagrange et al., 1998; Renfrow et al., 2004). The last 3-helices of each CLR comprise a typical HTH DNA-binding motif (Figure 9A). HTH motifs are comprised of H1-T1-H2-T2-H3 (H for helix; T for turn). Characteristically, H1 braces H2 and H3. H2 is generally a short helix. The N-terminus of H3 penetrates the major groove of DNA and makes most sequence-specific contacts. Figure 9A emphasizes the typical DNA contacts of HTH2 of TFB to the BREup (TFB-recognition element upstream of TATA) of the archaeal promoter. Figure 9B is a more complex image that includes TBP and CLR1 and CLR2 of TFB. H3 of CLR1 and CLR2 interacts with the major groove of DNA at BREdown and BREup. TFE is another GTF in Archaea that does not make extensive sequence-specific contacts to DNA (Blombach et al., 2015). In Bacteria, TBP and TFE appear to have been lost in evolution. The TFB C-terminal CLR/HTH repeats appear to have been duplicated and modified in evolution to generate bacterial σ factors.

**FIGURE 10:**
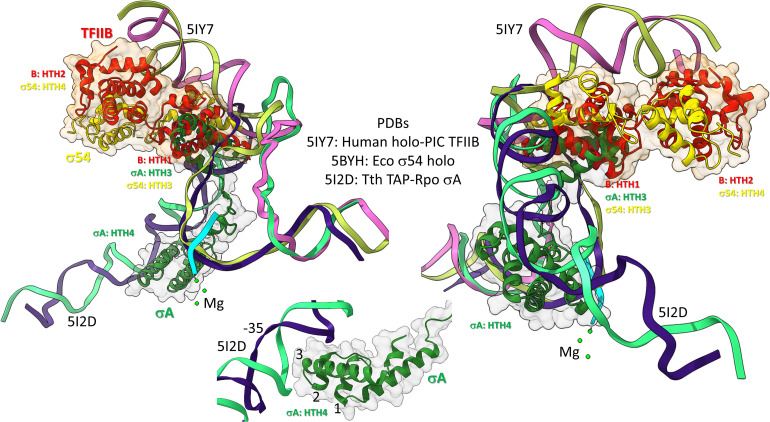
Bacterial σ factors and human TFIIB are homologs. Two views and one detail are shown. Two initiation complexes (human and *Thermus thermophilus*) and a σ54 holoenzyme structure (*Escherichia coli*) were overlaid. σA HTH3, σ54 HTH3, and TFIIB (B) HTH1 overlay at the upstream edge of the transcription bubble. σ54 HTH4 and TFIIB HTH2 partly overlay upstream (i.e., BREup). The detail is of σA HTH4 showing characteristic HTH contacts to the promoter –35 region. RNA is cyan. Mg is green. Upstream DNA strands are labeled: 5IY7: (pink) non-template; (yellow) template; and 5I2D: (green) non-template; (blue) template.

Bacterial σ factors are homologs of TFB (Iyer and Aravind, 2012; Burton, 2014; Burton and Burton, 2014; Burton et al., 2016; Figure 10). This idea was first postulated by Aravind and co-workers, based on the similarities of HTH units. Similarly to TFB, σ factors were initially strings of HTH units. For instance, σA appears to be derived from 4-HTH units (HTH1-4). We posit that σA was derived from duplication of the TFB C-terminus CLR/HTH units. σ54, by contrast, might be derived from 6–7 (or possibly 8) HTH units. σ54 might have resulted from early duplication of σA. The more N-terminal HTH units in both σA and σ54 are more degenerate, and, therefore, less recognizable. Here, we consider the four most C-terminal HTH units, which are in common comparing σA and σ54, and number them 1→4, from the N-terminal end, so HTH4 is the most C-terminal σ HTH unit. TFB, by contrast, includes two HTH units, numbered HTH1 and HTH2, C-terminal to an N-terminal Zn finger domain. So, HTH4 in σA and σ54 corresponds to HTH2 in TFB. HTH3 in σA and σ54 corresponds to HTH1 in TFB. The concept of σ and TFB homology is necessary to consider archaeal and bacterial divergence and the evolution and divergence of promoters.

To further support the homology of σ factors and TFB, we prepared overlays of initiation complexes from bacterial and human systems (Figure 10). Human TFIIB is a close homolog of archaeal TFB. RNAP and other GTFs were removed from the image to attempt simplification. Figure 10 is an overlay of three structures: (1) a human preinitiation complex (PDB 5IY7) (He et al., 2016), (2) a bacterial σA early initiation complex, with a short RNA (PDB 5I2D) (Feng et al., 2016), and (3) a bacterial σ54 holoenzyme (PDB 5BYH) (Yang et al., 2015). Because the image is somewhat busy, two views and a detail view are shown. TFIIB HTH1, σA HTH3 and σ54 HTH3 co-localize at the upstream end of the transcription bubble. TFIIB HTH2 and σ54 HTH4 partly overlay in the upstream DNA region. By contrast, σA HTH4 follows the diverging trajectory of the upstream DNA to which HTH4 binds at the −35 promoter region (detail image). Notice that σA HTH4 makes typical HTH contacts to the −35 region of the bacterial promoter (Figure 10; detail image), just as TFB makes typical HTH contacts to BREup and BREdown (Figure 9). We conclude from the overlay of these structures that HTH4 and HTH3 of bacterial σ factors correspond to HTH2 and HTH1 of human TFIIB (Iyer and Aravind, 2012; Burton, 2014; Burton and Burton, 2014; Burton et al., 2016).

### Promoter-Specific Regulatory HTH Factors

We speculate that GTFs TBP and TFB may have been present at LUCA as part of the earliest mechanisms for opening and managing DNA templates. In Archaea and Bacteria, many promoter-specific transcription factors are dimeric HTH or winged-HTH (HTH factors with β-sheet “wings”) factors (Aravind et al., 2005; Iyer and Aravind, 2012). These promoter-specific HTH factors may somehow have been derived by simplification of the CLR domains of TFB (5-α-helix bundles), followed generally by homodimerization. We note that bacterial σ factor HTH units are simplified from the TFB 5-helix CLR formats, from which σ factors appear to be derived (Iyer and Aravind, 2012; Burton and Burton, 2014). The HTH motif was, therefore, a core founding feature in Archaea and Bacteria of early evolution of both transcriptional GTFs (TFB and σ) and regulatory (HTH and winged-HTH factors) mechanisms.

### Evolution of Archaeal and Bacterial Promoters

**FIGURE 11:**
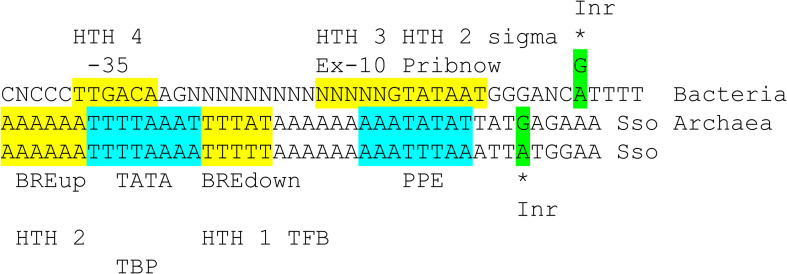
Comparison of bacterial σA promoters and archaeal promoters from *Sulfolobus solfataricus* (Sso; an ancient Archaea). See the text for details. Inr for initiator element.

A model for the divergence of archaeal and bacterial promoters is described (Figure 11). Because of the long passage of time, we are not certain that all aspects of a core promoter model can precisely be stated. The model is presented in order to provide a simple possible narrative that may stimulate more sophisticated bioinformatics approaches to this problem than we were able to do. Also, the model is based partly on our opinion that Archaea is most similar to LUCA, that Bacteria are more derived and that Bacteria evolved from Archaea (Battistuzzi et al., 2004; Marin et al., 2017; Lei and Burton, 2020; Long et al., 2020). There are reasons to consider this idea. A recent paper indicated that LUCA was most similar to Archaea, and that Bacteria were derived from Archaea. tRNAs and tRNAomes (all the tRNAs for an organism) are simpler and more similar to the primordial tRNA sequence in Archaea (Pak et al., 2018; Kim et al., 2019; Lei and Burton, 2020). Also, aminoacyl-tRNA synthetases and the genetic code are simpler to model in Archaea than in Bacteria, indicating that Archaea are more similar to LUCA than Bacteria.

Figure 11 compares a bacterial σA promoter and its GTF contacts and an archaeal promoter and its GTF contacts. The bacterial promoter shows sequences characteristic of a strong promoter with multiple contacts to different regions of σA. Bacteria lack TBP and TFE, which we posit may have been lost during bacterial divergence. Bacteria include RNase HIII that includes a TBP fold (Brindefalk et al., 2013), however, possibly indicating that Bacteria had TBP as a transcription factor from Archaea and then lost TBP in evolution, as we propose. According to the structural overlay (Figure 10), bacterial σA HTH4 and HTH3 correspond to archaeal TFB HTH2 and HTH1 (Iyer and Aravind, 2012; Burton, 2014; Burton and Burton, 2014; Burton et al., 2016). Bacterial σA HTH4 contacts the −35 region of promoters [i.e., (-34)-TTGACA-(-29)]. Archaeal TFB HTH2 contacts the BREup (TFB-recognition element upstream of the TATA-box). TBP binds the 8-nt TATA-box [i.e., (-30)-TTTTAAAA-(-23) in *Sulfolobus solfataricus*] (Ao et al., 2013), but TBP is missing in Bacteria. Bacterial σA HTH3 partly contacts the Extended −10 sequence in double-stranded DNA, found in some promoters, and then resides on double-stranded DNA at the upstream edge of the transcription bubble, as the promoter opens (Figure 10). Archaeal TFB HTH1 contacts the BREdown (TFB-recognition element downstream of the TATA-box) (an A/T-rich sequence downstream from TATA in *Sulfolobus solfataricus*) (Figure 9B). After promoter opening, TFB HTH1 occupies double-stranded DNA just upstream of the transcription bubble (Figure 10).

The Promoter-Proximal Element (PPE) is an A/T-rich sequence in *Sulfolobus solfataricus* promoters upstream of the transcription start [i.e., ∼(−11)-AATATTAA-(−4)] (Ao et al., 2013). To us, the PPE resembles a TATA-box and may be derived from one. The PPE appears to be positioned similarly to the bacterial Pribnow box [i.e., (−12)-TATAAT-(−7)] and is similar in sequence. We, therefore, posit that the Pribnow box of bacterial promoters may be derived from an archaeal PPE sequence. Notably, the Pribnow box is recognized by σA HTH2, which is a modified HTH with interesting characteristics. The σA HTH2 opens the bacterial promoter by flipping bases. A(−11) is first flipped out followed by T(−7), leading to promoter opening (Feklistov and Darst, 2011; Feklistov et al., 2014; Boyaci et al., 2019).

Archaeal promoters typically have an initiator sequence surrounding + 1, the transcription start (Ao et al., 2013). Many promoters have (−1)-TATG-(+3). In this case, no 5′-untranslated sequence may be present in the mRNA, which may initiate translation at (+1)-AUG-(+3). (−1)-TGAG-(+3) is also common. In this case, translation generally initiates at a downstream AUG. The initiator element is thought to be recognized directly by RNAP. Bacteria also have an initiator sequence (Cassiano and Silva-Rocha, 2020). Both Archaea and Bacteria utilize ribosome attachment sequences (i.e., AGGA) on some mRNAs with a corresponding interaction sequence near the 3′-end of 16S rRNA (i.e., UCCU).

### Interactions of DPBB Loops With GTFs

One hypothesis might be that multi-subunit RNAP DPBB loops that include inserts contact GTFs in a domain-specific fashion. The idea underlying this hypothesis is that DPBBs form the catalytic center and hold the active site Mg1 and Mg2. The RNAP active site is deeply sequestered within the RNAP core, limiting access to the catalytic center. Inserts in the DPBB loops might allow GTFs binding closer to the RNAP periphery to communicate with catalytic functions. Because archaeal GTFs and TFB are so different from bacterial σ factors, TFB and σ might be expected to interact with DPBB loops with distinct, domain-specific inserts.

**FIGURE 12:**
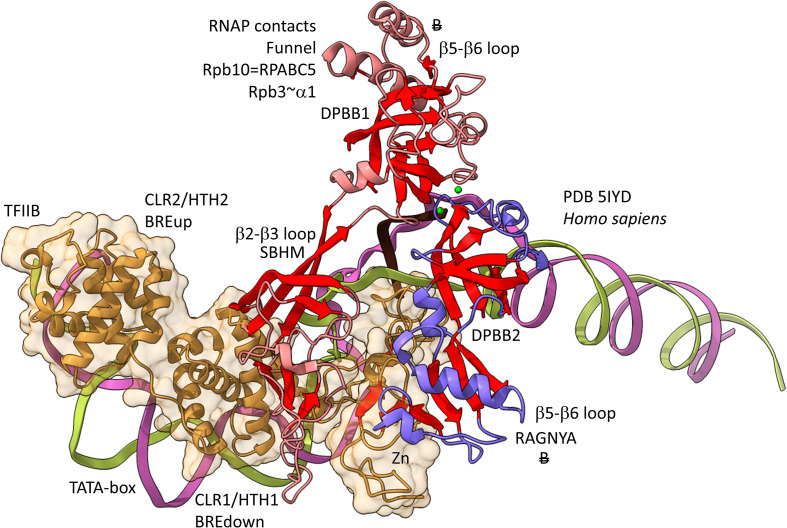
Archaea/Eukaryote-specific contacts of TFB/TFIIB with DPBB insert loops. β-sheets are red. Other features of Rpb1 are blue and Rpb2 are light red. TFIIB is orange with transparent space-filling representation. “B” with double strike through indicates a contact specific to Archaea and not found or very different in Bacteria.

**FIGURE 13:**
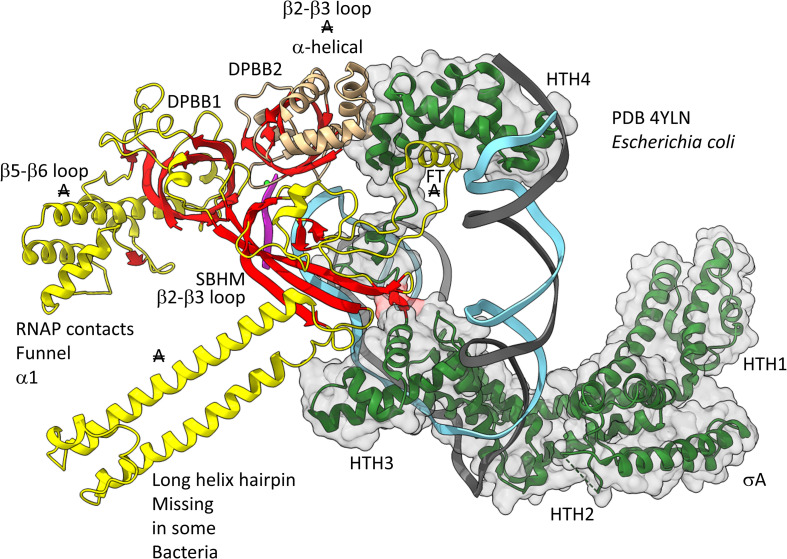
Bacteria-specific contacts of σA with DPBB insert loops. β-sheets are red. Other β′ features are beige, and β features are yellow. σA is green with transparent space-filling representation. FT for flap tip helix. “A” with double strike through indicates a feature found in Bacteria but very different or not identified in Archaea.

Figures 12, 13 show domain-specific functional contacts of DPBB loops with GTFs. Figure 12 shows a simplified view of a human preinitiation complex (PDB 5IYD) (He et al., 2016). Most of the factors in the structure have been removed to simplify the image. The human DPBB1 SBHM (β2-β3 insert) contacts TFIIB HTH1/CLR1 located at the upstream edge of the transcription bubble. Interestingly, the human DPBB2 RAGNYA β2-β3 insert, specific for Archaea and Eukaryotes, contacts the N-terminal Zn finger of TFIIB. In Figure 13, a detail of the *Escherichia coli* RNAP initiation complex is shown (PDB 4YLN) (Zuo and Steitz, 2015). Bacterial σA HTH3, at the upstream end of the transcription bubble, contacts the SBHM. Thus, homologous GTFs in Archaea (TFB) and Bacteria (σA) make domain-specific contacts to their domain-specific SBHMs. In Bacteria, the flap tip helix is an extension of the SBHM that contacts the σA HTH4, bound to the −35 promoter region. Interestingly, the *Escherichia coli* RNAP SBHM includes a long helix hairpin motif as an insert, missing in Archaea and many Bacteria (i.e., missing in *Thermus thermophilus*, an ancient Bacteria). The long helix hairpin insert contacts σA HTH3 in the initiating complex. The DPBB2 β2-β3 insert in *Escherichia coli* RNAP is an α-helical motif that substitutes for the very different RAGNYA insert in Archaea, which contacts the N-terminal Zn motif in TFIIB (Figure 12). The corresponding DPBB2 β2-β3 α-helical insert in Bacteria makes domain-specific contacts to αA HTH4, bound at the −35 promoter region (Figure 13).

**FIGURE 14:**
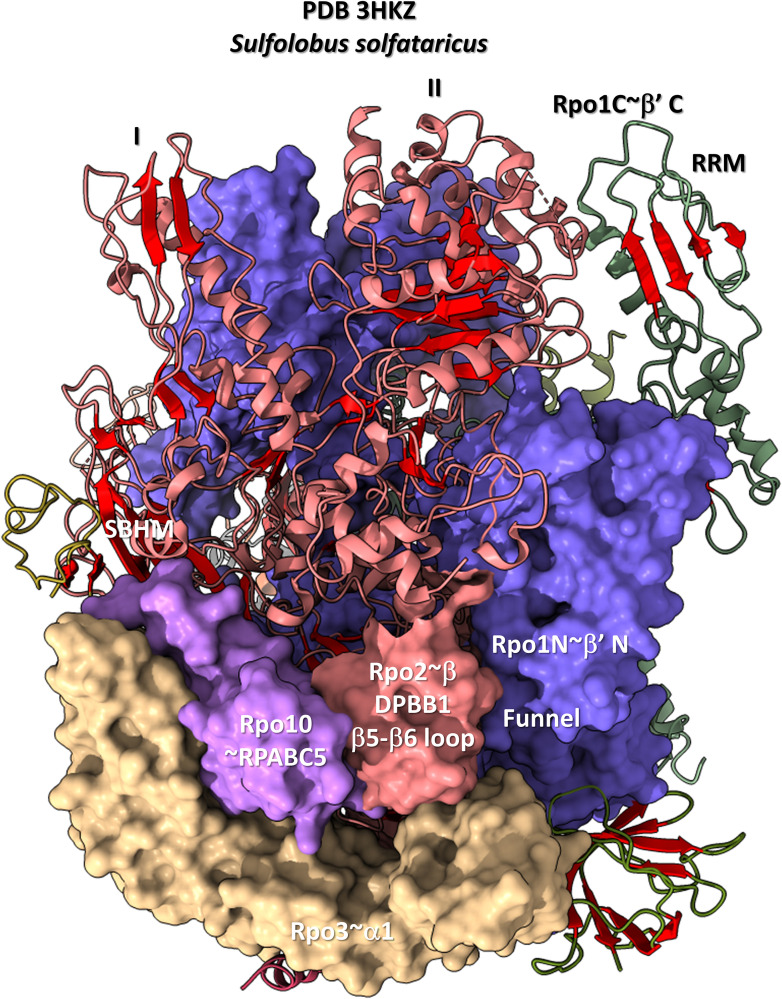
The DPBB1 β5-β6 loop (space-filling representation) contacts RNAP. In Archaea, the DPBB1 β5-β6 loop contacts the Rpo1N (homolog of β′ in Bacteria) funnel, the Rpo2 (homolog of β in Bacteria) N-terminal domain (lobe II), Rpo3 (homolog of α1 in Bacteria) and Rpo10 (homolog of RPABC5 in Eukarya). The SBHM contacts lobe I of the N-terminal Rpo2 domain and Rpo3.

The DPBB1 β5-β6 insert shows some homology in Archaea and Bacteria but, also, significant domain-specific character, so we attempted to identify a GTF that might contact this region. We were unsuccessful. So far as we can discern, the β5-β6 DPBB1 inserts in Archaea and Bacteria make domain-specific contacts to other regions of RNAP (Figure 14). In Archaea, the β5-β6 DPBB1 insert contacts: (1) the Rpo1N funnel (A′; homolog of β′ in Bacteria); (2) Rpo10 (N; homolog of RPABC5 in Eukarya); and (3) Rpo3 (C; homolog of α1 in Bacteria). In Bacteria, the β5-β6 DPBB1 insert makes similar domain-specific contacts to RNAP (not shown).

During transcription elongation, TFB and σ factors cycle off RNAP and are replaced by the elongation factor homologs Spt5/Spt4 in Archaea and NusG in Bacteria (Werner, 2012; Blombach et al., 2013; Hartzog and Fu, 2013; Tomar and Artsimovitch, 2013; Yakhnin and Babitzke, 2014; Wang and Artsimovitch, 2020). These elongation factors occupy approximately the same positions on RNAP as HTH2 and HTH3 of bacterial σA (not shown). These elongation factors, therefore, make domain-specific contacts to the SBHM of their DPBB1 (i.e., see PDB 5TBZ) (Liu and Steitz, 2017). Contacts to GTFs are also specific to the initiation and elongation phases of the transcription cycle. For instance, in Bacteria, the flap tip helix contacts σA during initiation (Figure 13) but does not contact NusG during elongation.

## Divergence of Archaea and Bacteria

Evolution of life on Earth appears to be a simple outline with overwhelming detail. According to our view, pre-life evolved to LUCA, which we interpret as an ancient Archaea. Archaea diverged to generate Bacteria, which became a more flexible and, in many ways, more successful prokaryotic domain, restricting Archaea somewhat to the margins (i.e., to extremophile environments). Multiple Archaea and Bacteria fused to form Eukaryotes, which have occupied many new niches on Earth (Forterre, 2015; Castelle and Banfield, 2018; Eme et al., 2018). Ancient Archaea, therefore, are very similar to LUCA. Bacteria are more innovated than Archaea and more derived evolutionarily. Because of their mitochondria and complex genomes and development, Eukaryotes have many new capacities lacking in Archaea and Bacteria. We refer to the splitting of the archaeal and bacterial domains as “the great divergence,” and we consider this event to be one of the most important advances in evolution of life as we know it on Earth.

There are several defining differences comparing Archaea and Bacteria: i.e., (1) evolution of TFB (Archaea) versus σ factors (Bacteria); (2) utilization of DNAPs PolD and PolB (Archaea) versus PolC (Bacteria) (Koonin et al., 2020), and (3) archaeal versus bacterial membranes (Lane and Martin, 2012; Lane, 2020). Above, we have discussed the divergence of archaeal and bacterial GTFs and promoters in some detail. We consider modifications of bacterial transcription systems to be fundamental and possibly the founding difference in the great divergence of Bacteria from Archaea. For instance, evolution of bacterial σ factors appears to have driven the simplification and divergence of bacterial RNAPs from archaeal ancestors.

## Author Contributions

All authors listed have made a substantial, direct and intellectual contribution to the work, and approved it for publication.

## Conflict of Interest

The authors declare that the research was conducted in the absence of any commercial or financial relationships that could be construed as a potential conflict of interest.

## Abbreviations

BH: bridge helix
BRE: TFB-recognition element
CLR: cyclin-like repeat (TFB HTH domains)
DNAP: DNA polymerase
DPBB: double- Ψ - β -barrel
HTH: helix-turn-helix
InR: initiator element
LUCA: last universal (cellular) common ancestor
Pfu: *Pyrococcus furiosis*
Pol: DNA polymerases (i.e. PolA, PolB, PolC, and PolD)
PPE: promoter-proximal element
RNAP: RNA polymerase
RRM: RNA-recognition motif
SBHM: sandwich barrel hybrid motif
Sso: *Sulfolobus* solfataricus
TBP: TATA-box binding protein
TFB: transcription factor B
TFE: transcription factor E
TIM: triose phosphate isomerase
TL: trigger loop.
